# Zoonotic pathogens survey in free-living long-tailed macaques in Thailand

**DOI:** 10.1080/23144599.2022.2040176

**Published:** 2022-02-28

**Authors:** Supakarn Kaewchot, Siriporn Tangsudjai, Ladawan Sariya, Chalisa Mongkolphan, Aeknarin Saechin, Rattana Sariwongchan, Natanon Panpeth, Salintorn Thongsahuan, Parut Suksai

**Affiliations:** a Department of National Parks, Wildlife and Plant Conservation, Bangkok, Thailand; b The Monitoring and Surveillance Center for Zoonotic Diseases in Wildlife and Exotic Animals, Faculty of Veterinary Science, Mahidol University, Nakhon Pathom, Thailand

**Keywords:** Hepatitis B virus, long-tailed macaque, *Macaca fascicularis*, *Plasmodium inui*, simian foamy virus

## Abstract

Long-tailed macaques (*Macaca fascicularis*) are known to harbour a variety of infectious pathogens, including zoonotic species. Long-tailed macaques and humans coexist in Thailand, which creates potential for interspecies pathogen transmission. This study was conducted to assess the presence of B virus, *Mycobacterium* spp., simian foamy virus (SFV), hepatitis B virus (HBV), and *Plasmodium* spp. in 649 free-living Thai long-tailed macaques through polymerase-chain reaction. DNA of SFV (56.5%), HBV (0.3%), and *Plasmodium* spp. (2.2%) was detected in these macaques, whereas DNA of B virus and *Mycobacterium* spp. was absent. SFV infection in long-tailed macaques is broadly distributed in Thailand and is correlated with age. The HBV sequences in this study were similar to HBV sequences from orangutans. *Plasmodium* spp. DNA was identified as *P. inui*. Collectively, our results indicate that macaques can carry zoonotic pathogens, which have a public health impact. Surveillance and awareness of pathogen transmission between monkeys and humans are important.

## 1. Introduction

The most infectious diseases are zoonoses, which can be transmitted from animals to humans [1]. Zoonotic diseases are caused by viruses, bacteria, fungi, parasites, and prions [1] and pose a serious threat to public health and economy [2]. Wildlife is a major source of zoonotic disease [2]. Non-human primates (NHPs) are a reservoir host of several infectious diseases [2–4]. Of the 25 important zoonotic diseases in humans, 5 originate from NHPs [5]. NHPs can spread pathogens to humans via body fluids, contaminated food or water, and insect vectors. NHPs can carry several pathogens that lead to human infections, such as B virus, *Mycobacterium* spp., simian foamy virus (SFV), hepatitis B virus (HBV), and *Plasmodium* spp. The seropositivity rate for the B virus in adult captive macaques is high and without clinical signs [6]. B virus infections in humans are usually transmitted by macaques. Although the incidence of B virus infection in humans is low, the mortality is high (>70%) [6]. *Mycobacterium* spp. is another pathogen that poses a serious problem for public health worldwide [7]. Macaques are the most susceptible species to *Mycobacterium* infections [7]. SFV is a retrovirus that is found in both Old and New World NHP [8], especially macaques [9]. Approximately 1–5% of the humans in close contact with NHPs were found to be infected with SFV [10]. HBV is mainly reported in Old World NHPs, including the long-tailed macaques [11,12]. This virus is a global health problem with a high prevalence in humans [13]. *Plasmodium* spp. cause vector-borne diseases and their occurrence in NHPs results in morbidity and mortality in humans [14]. Three simian malaria species (*P. cynomolgi, P. knowlesi*, and *P. inui*) are natural parasites in Southeast Asian macaques that can infect humans [14–16].

Thailand supports 15 native NHP species [17], with a larger population of long-tailed macaques (*Macaca fascicularis*) [18,19]. Long-tailed macaque habitats are located throughout Thailand [18,19]. Most habitats are shared with human communities due to macaque overcrowding, the adaptation of macaque behaviour, the sizes of human communities, and the nature of human activities [18,19]. Conflicts between these NHP and humans are common [18,19]. Such contact creates opportunities for cross-transmission of pathogens between macaques and humans. This study This study was carried out to investigate B virus, *Mycobacterium* spp., SFV, HBV, and *Plasmodium* spp. infection in free-living long-tailed macaques inhabiting urban areas in Thailand.

## 2. Materials and methods

### 2.1. Ethical statement

The protocol of this study was approved by the Animal Care and Use Committee, Faculty of the Veterinary Science, Mahidol University (FVS-MU-IACUC) (Ethical approval No. MUVS-2020-02-05).

### 2.2. Sample collection and DNA extraction

**Figure 1.**
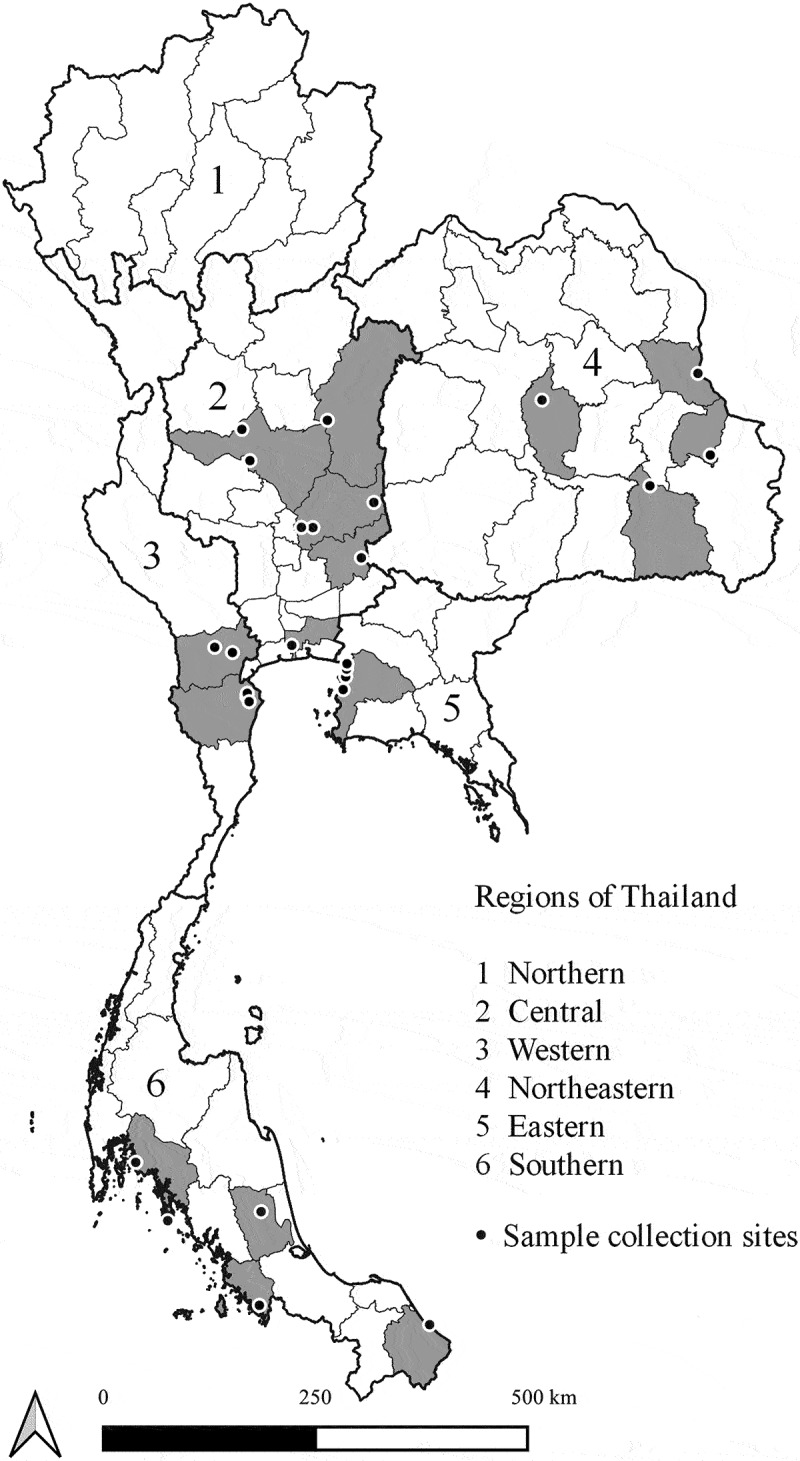
Location of collection sites of long-tailed macaques in Thailand. The map was created using QGIS version 3.8.3-Zanzibar, a free and open source geographic information system.

Accessing sample size, Slovin’s formula was used to calculate the number of long-tail macaques in Thailand for this study. A total of 649 free-living, long-tailed macaques were captured from different locations in 26 urban areas in 5 regions in Thailand (Figure 1). Sites and samples were distributed throughout the country, including eight sites in the Central (n = 206), four sites in the Eastern (n = 100), five sites in the Western (n = 125), five sites in the Southern (n = 118), and four sites in Northeastern (n = 100) regions (Table 1). Blood and oropharyngeal swabs were obtained from 649 anesthetized animals under tiletamine HCl-zolazepam (3 mg/kg) -xylazine (0.5 mg/kg). After the animals fully recovered, returning them to their homeland. Animals were classified as adults and sub-adults as judged by body size, genital organs, teeth, and muscular development. Samples were collected between March and September 2019. DNA was extracted from blood and swab samples using DNeasy Blood & Tissue Kit (QIAGEN, Germany), following the manufacturer’s instructions. Animals consisted of 446 males (147 sub-adults, 299 adults) and 203 females (81 sub-adults, 122 adults) (Table 1). Macaques were judged healthy by physical examination.

**Table 1.** The number of collected long-tailed macaque samples within different geographical regions

Thailand Region	Site	Male			Female		
		Sub-adult	Adult	Total	Sub-adult	Adult	Total
Central	8	35	104	139	28	39	67
Eastern	4	36	42	78	12	10	22
Western	5	29	77	106	7	12	19
Southern	5	33	45	78	25	15	40
Northeastern	4	14	31	45	9	46	55
Total		147	299	446	81	122	203

### 2.3. Molecular detection of pathogens

All macaques were screened for several pathogens, including B virus, *Mycobacterium* spp., SFV, HBV, and *Plasmodium* spp., by polymerase-chain reaction (PCR). DNA from oropharyngeal swabs was investigated in the presence of SFV, B virus, and *Mycobacterium* spp. while the extracted DNA from blood collected into EDTA-containing tubes was examined for HBV and *Plasmodium* spp.

The status of B virus samples was assessed with the glycoprotein G (*G*) gene by real-time PCR [20]. The identification of *Mycobacterium* spp. was carried out through a multiplex PCR with three primer sets [21]. These primers target insertion sequences *6110* (IS*6110*), 32-kD alpha protein (32-kDa), and MTP40 species-specific protein (*mtp40*) genes, which are specific for the *M. tuberculosis* complex, *Mycobacterium* spp., and *M. tuberculosis*, respectively [21]. To detect SFV, Nested PCR was used to amplify the polymerase (*pol*) gene of the proviral DNA [22]. HBV was screened using a duplex real-time PCR based on the surface (*S*) and core (*C*) genes [23]. Positive HBV samples were identified with nested PCR to amplify *S* as previously documented [24]. For *Plasmodium* spp., a real-time PCR was conducted [25]. Macaques positive for *Plasmodium* infection were screened for four human malaria species (*P. falciparum, P. vivax, P. ovale*, and *P. malariae*) and five simian malaria species (*P. knowlesi, P. cynomolgi, P. coatneyi, P. inui*, and *P. fieldi*) using nested PCR [26,27]. Species in the genus *Plasmodium* were identified in the first round of PCR, and individual species were identified in the second round. Both real-time and nested PCRs target the 18S small subunit ribosomal RNA (18S rRNA) genes. Specific primer sets and PCR conditions for this study were previously described [20–27] (Table 2).

**Table 2.** Sequences of primers and probes for pathogens detection in this study

Primer/Probe	Sequences (5’-3’)	Annealing Temperature (°C)	Product size (bp)	Reference
B virus detection				
gG_BV323F	TGGCCTACTACCGCGTGG	60	123	[20]
gG_BV446R	TGGTACGTGTGGGAGTAGCG			
gG_BV403P	FAM-CCGCCCTCTCCGAGCACGTG-TAMRA			
*Mycobacterium* spp. detection				
*M. tuberculosis* complex				
IS5-F	CGGAGACGGTGCGTAAG	70	984	[21]
IS6-R	GATGGACCGCCAGGGCTTGC			
*Mycobacterium* spp.				
MT1	TTCCTGACCAGCGAGCTGCCG	70	506	
MT2	CCCCAGTACTCCCAGCTGTGC			
*M. tuberculosis*				
PT1	CGGCAACGCGCCGTCGGTGG	70	396	
PT2	CCCCCCACGGCACCGCCGGG			
SFV detection				
SFV_pol_F1	GTGGNAAGGTGGAAAGGA AAA ATAGTGANA	45	227	[22]
SFV-pol_R1	NTANAGANNNNCNAATTTCCTGTAAAAGAGA			
SFV_pol_F2	NGTNGGNNGNCCTNCNAAGTGGTATGA	47	153	
SFV_pol_R2	NAANTCAAGTGTATCNNNNTTTGCAAANGG			
HBV detection				
HBV-C-F	TTCCGGAAACTACTGTTGTTAGAC	55	125	[23]
HBV-C-R	ATTGAGATTCCCGAGATTGAGA			
HBV-C-P	FAM-CCCTAGAAGAAGAACTCCCTCGCCTC-BHQ1			
HBV-S-F	GATGTGTCTGCGGCGTTTTA	55	91	
HBV-S-R	GCAACATACCTTGATAGTCCAGAAGAA			
HBV-S-P	Cy5-CCTCTICATCCTGCTGCTATGCCTCA-BHQ2			
HBV confirmation				
HBV-S1	CATCAGGAYTCCTAGGACCCCT	55	238	[24]
HBV-S5	GAGGCATAGCAGCAGGATGMAGAGG			
HBV-S3	CGTGTTACAGGCGGKGTKTTTCTTGT	55	206	
HBV-S6	ATGATAAAACGCCGCAGACACATC3			
*Plasmodium* spp. detection				
Plasmo-F	GCTCTTTCTTGATTTCTTGGATG	60	99	[25]
Plasmo-R	AGCAGGTTAAGATCTCGTTCG			
Plasmo-P	FAM-ATGGCCGTTTTTAGTTCGTG-TAMRA			
Human and simian malaria species identification				
*Plasmodium* spp.				
rPLU5	CCTGTTGTTGCCTTAAACTTC	55	1,100	[26]
rPLU6	TTAAAATTGTTGCAGTTAAAACG			
*P. falciparum*				
rFAL1	TTAAACTGGTTTGGGAAAACCAAATATATT	55	205	
rFAL2	ACACAATGAACTCAATCATGACTACCCGTC			
*P. vivax*				
rVIV1	CGCTTCTAGCTTAATCCACATAACTGATAC	55	120	
rVIV2	ACTTCCAAGCCGAAGCAAAGAAAGTCCTTA			
*P. ovale*				
rOVA1	ATCTCTTTTGCTATTTTTTAGTATTGGAGA	55	800	
rOVA2	GGAAAAGGACACATTAATTGTATCCTAGTG			
*P. malariae*				
rMAL1	ATAACATAGTTGTACGTTAAGAATAACCGC	55	144	
rMAL2	AAAATTCCCATGCATAAAAAATTATACAAA			
*P. knowlesi*				
Pmk8	GTTAGCGAGAGCCACAAAAAAGCGAAT	60	153	[27]
Pmk9r	ACTCAAAGTAACAAAATCTTCCGT			
*P. cynomolgi*				
CY2F	GATTTGCTAAATTGCGGTCG	61	137	
CY4R	CGGTATGATAAGCCAGGGAAGT			
*P. coatneyi*				
PctF1	CGCTTTTAGCTTAAATCCACATAACAGAC	61	504	
PctR1	GAGTCCTAACCCCGAAGGGAAAGG			
*P. inui*				
PinF2	CGTATCGACTTTGTGGCATTTTTCTAC	61	479	
INAR3	GCAATCTAAGAGTTTTAACTCCTC			
*P. fieldi*				
PfldF1	GGTCTTTTTTTTGCTTCGGTAATTA	62	421	
PfldR2	AGGCACTGAAGGAAGCAATCTAAGAGTTTC			

### 2.4. Phylogenetic analysis

*S* amplicons of HBV are sequenced and used for phylogenetic tree construction. The tree was generated by bootstrap analysis with 1,000 replicates. Phylogenetic relationships of various HBV strains were constructed using the ClustalW alignment and the maximum likelihood with the MEGA 7 software [28]. Distances were evaluated with the Kimura two-parameter model assuming a Gamma distribution. The reference sequences for HBV for comparison were obtained from GenBank NCBI (http://www.ncbi.nlm.nih.gov/genbank).

### 2.5. Nucleotide sequence accession numbers

Partial *S* gene sequences of HBV detected from the Central (MW2536/62) and the Northeastern (MW2227/62) regions were deposited on GenBank with accession no. OL711942 and OL711943, respectively.

### 2.6. Statistical analysis

Statistical relationships between positive results and status data, including sex and age of free-living NHPs, were analysed using Pearson chi-square tests using SPSS 26 for Windows (SPSS Inc., USA). Statistical significance was indicated at a *P*-value of <0.05.

## 3. Results

A total of 649 long-tailed macaques native to Thailand were screened for B virus, *Mycobacterium* spp., SFV, HBV, and *Plasmodium* spp. using specific PCR-based methods. No macaque samples were positive for B virus or *Mycobacterium* spp. DNA from SFV, HBV, and *Plasmodium* spp. was recovered (Table 3). SFV was encountered in over half of the oropharyngeal swabs (56.5%, 367/649) and was present at every sampling site. SFV was identified in 56.5% (252/446) of male and 56.7% (115/203) of female macaques. Further, SFV-positive males included 63.2% (189/299) of adults and 42.9% (63/147) of sub-adults. Similarly, 61.5% (75/122) and 49.4% (40/81) of positive females were adults and sub-adults, respectively. Association analysis showed no significant difference between the positive results for SFV and sex (*P* = 0.972), but a significant relationship was observed between SFV and age (*P* < 0.001). Furthermore, the number of SFV-positive cases in the female group was not significantly associated with age (*P* = 0.089) but showed a significant relationship with age in the male group (*P* < 0.001).

**Table 3.** Age and sex distribution of zoonotic pathogens among Thai long-tailed macaques

		Number of samples (%)	Number of positive		
			SFV (%)	HBV (%)	Plasmodium spp. (%)
Sex	Male	446 (68.7)	252 (56.5)	2 (0.4)	10 (2.2)
	Female	203 (31.3)	115 (56.7)	0 (0)	4 (2)
	*P*-value		0.972	0.339	0.825
Age	Sub-adult	228 (35.1)	103 (45.2)	0 (0)	5 (2.2)
	Adult	421 (64.9)	264 (62.7)	2 (0.5)	9 (2.1)
	*P*-value		< 0.001	0.297	0.963
Total		649	367 (56.5)	2 (0.3)	14 (2.2)

**Figure 2.**
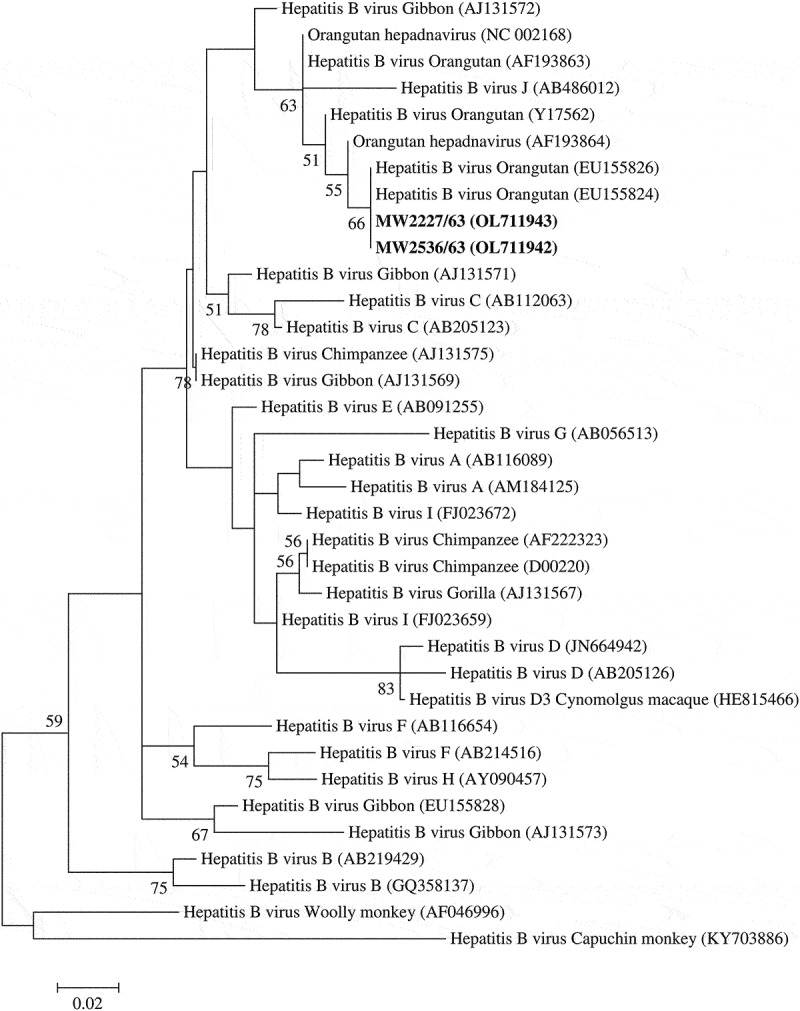
Phylogenetic tree based on *S* gene of HBV was constructed by the Maximum likelihood method. The bold is the positive samples in this study. The phylogenetic tree illustrated sequence relationships among positive samples, genotypes of human HBV, and sequences of NHP HBV. Bootstrap analysis was calculated with 1,000 replicates. Percentages of bootstrap values were displayed on the nodes of the tree, although values below 50% were excluded.

Out of the 649 long-tailed macaques, 2 (0.3%) were HBV positive by duplex real-time PCR. The positive HBV samples were adult males from Central (n = 1) and Northeastern (n = 1) regions. These animals were co-infected with SFV. Further, these positive samples showed 100% similarity to sequences of HBV found in orangutans from Thailand using a BLASTn search. Positive sequences clustered with the HBV from orangutans in a phylogenetic tree based on the HBV *S* gene (Figure 2).

*Plasmodium* spp. DNA was identified in 14 of 649 (2.2%) blood samples from 2 sites of Central and 2 sites of Southern areas. Of the 14 *Plasmodium*-positive animals, 9 were co-infected with SFV. *Plasmodium* was detected in both males (n = 10) and females (n = 4). Seven and three positive males were adults and sub-adults, respectively. The same number of positive female adults and sub-adults was observed. All *Plasmodium*-positive samples were identified as *P. inui*. Positive rates were not statistically significantly different for sex (*P* = 0.825) or age (*P* = 0.963).

## 4. Discussion

Most habitats of long-tailed macaques in Thailand overlap with human communities [18,19]. This overlap creates an opportunity for cross-transmission of pathogens between macaques and humans. Thus, long-tailed macaques from colonies near communities or tourist attractions were examined in the present study.

None of the 649 oropharyngeal swabs collected in this study were positive for the B virus using real-time PCR. B virus DNA was previously identified among long-tailed macaques in Malaysia (39.3%) [29], Japanese macaques in Japan (10%) [30], and rhesus macaques in the USA (2.5%) [31]. Currently, shedding of the B virus has not been reported in Thai macaques. These pathogens are typically found in macaques, but the shedding frequency remains unclear [6]. The shedding frequency of the B virus may not be high [6].

All macaques in this study were negative for *Mycobacterium* spp. infection. This pathogen can be found in both captive and free-ranging NHPs [32,33]. The most susceptible species are the Old World monkeys, especially rhesus macaque [7,34]; the long-tailed macaque is less susceptible [34]. Our finding contrasts with an earlier study that showed 50% (5/10) incidence in Thai long-tailed macaques [32]. This disparity might be attributed to the health status of macaques because long-tailed macaques included in the previous study had a history of respiratory symptoms [32].

Proviral DNA from SFV in oropharyngeal swabs (56.5%) was amplified and found to be highly prevalent, consistent with the previous information on SFV prevalence in NHPs [8,10,35], particularly in Asian macaques [4,9,36,37]. Our results support a high incidence of SFV antibodies in free-range long-tailed macaques in Thailand (81%) [9]. Altogether, SFV appears to be commonly circulated among long-tailed free-living macaques in Thailand. Such a result is consistent with Gibraltar [38], i.e. SFV prevalence depends on age but does not correlate with sex. In more detail, the increase in SFV prevalence depends on age in the male group (*P* < 0.001) but is not associated with the female group (*P* = 0.089). This finding demonstrated a result of social behaviours in which adult males have more aggression, fighting, and biting than sub-adult males and females. This behaviour is a route to transmission of SFVs.

Of the 649 blood samples from long-tailed macaques, 2 (0.3%) were positive for HBV with duplex real-time PCR. This HBV DNA positivity was lower than the previously reported incidence in sera and livers from Mauritius long-tailed macaques (25.8% and 42%, respectively) [11]. In Thailand, HBV DNA was found in 18.81% (19/101) of sera samples from captive gibbons [39]. Conversely, HBV infection in Thai long-tailed macaques has not been previously reported. The lower prevalence in our study might reflect the geographical distribution or type of sampled populations. Acute and chronic HBV infections in humans may present as asymptomatic or with severe signs [40]. None of the HBV-positive macaques in this study showed clinical signs. A previous study reported that NHPs with HBV infection can be asymptomatic carriers [12]. Nucleotide sequence and phylogeny analysis based on the *S* gene were used to identify HBV in this study. Positive sequences were similar to HBV sequences from captive orangutans in Thailand. In the phylogram, macaque sequences clustered with HBV of captive orangutans in Thailand. Long-tailed macaques and orangutans may be infected with the same HBV strains. The present finding contrast with the previous report that genotype D of HBV was identified in Mauritius long-tailed macaque [11].

Regarding *Plasmodium* spp. detection, *P. inui* was identified in all *Plasmodium*-positive samples, which accounted for 2.2% of all macaques examined in this study. *Plasmodium* spp., especially *P. inui*, is normally found in long-tailed macaques [27,41–43]. Positivity for *P. inui* in wild long-tailed macaques from the Southern region in Thailand was 5.05% (5/99) [43] and 3.59% (7/195) [42]. No correlation between infection and sex or age was observed in the study population. Similar *Plasmodium* infection incidence was reported in wild macaques in Malaysia [44]. *P. inui* has been reported to cause infection in humans naturally and experimentally [14–16]. Indeed, natural infection in humans is associated with no clinical signs and a very low number of parasites in blood [15].

The limitations of this study are that nucleotide sequencing and phylogenetic tree construction of SFV and *P. inui* were not performed because SFV and *P. inui* are generally found in Thai macaques [9,42,43,45] as well as natural infection with SFV and *P. inui* in humans is still very rare (1–5% for SFV and 2.82% for *P. inui*) and asymptomatic [10,15]. The public health burden of such pathogens needs to be further investigated in the future

### 5. Conclusion

In conclusion, SFV, HBV, and *Plasmodium* spp., were found in wild macaques, indicating that these NHPs may serve as a reservoir for zoonotic pathogens in Thailand. Awareness and surveillance of transmission of such pathogens between monkeys and humans is critical. The findings in this study provide information to government agencies for risky communication on zoonotic diseases in communities that share an interface with free-living macaques.

## References

[1] Lloyd-Smith JO, George D, Pepin KM, et al. Epidemic dynamics at the human-animal interface. Science. 2009;326(5958):1362–1367. 19965751 10.1126/science.1177345 PMC3891603

[2] Kruse H, Kirkemo AM, Handeland K. Wildlife as source of zoonotic infections. Emerg Infect Dis. 2004;10(12):2067–2072. 15663840 10.3201/eid1012.040707 PMC3323390

[3] Nakayima J, Hayashida K, Nakao R, et al. Detection and characterization of zoonotic pathogens of free-ranging non-human primates from Zambia. Parasit Vectors. 2014;7(1):490. 25358853 10.1186/s13071-014-0490-x PMC4221724

[4] Jones-Engel L, Engel GA, Heidrich J, et al. Temple monkeys and health implications of commensalism, Kathmandu, Nepal. Emerg Infect Dis. 2006;12(6):900–906. 16707044 10.3201/eid1206.060030 PMC3373059

[5] Wolfe ND, Dunavan CP, Diamond J. Origins of major human infectious diseases. Nature. 2007;447(7142):279–283. 17507975 10.1038/nature05775 PMC7095142

[6] Huff JL, Barry PA. B-virus (*Cercopithecine herpesvirus* 1) infection in humans and macaques: potential for zoonotic disease. Emerg Infect Dis. 2003;9(2):246–250. 12603998 10.3201/eid0902.020272 PMC2901951

[7] Ghodbane R, Drancourt M. Non-human sources of *Mycobacterium tuberculosis*. Tuberculosis (Edinb). 2013;93(6):589–595. 24119770 10.1016/j.tube.2013.09.005

[8] Meiering CD, Linial ML. Historical perspective of foamy virus epidemiology and infection. Clin Microbiol Rev. 2001;14(1):165–176. 11148008 10.1128/CMR.14.1.165-176.2001 PMC88968

[9] Jones-Engel L, Steinkraus KA, Murray SM, et al. Sensitive assays for simian foamy viruses reveal a high prevalence of infection in commensal, free-ranging Asian monkeys. J Virol. 2007;81(14):7330–7337. 17475645 10.1128/JVI.00343-07 PMC1933339

[10] Murray SM, Linial ML. Foamy virus infection in primates. J Med Primatol. 2006;35(4–5):225–235. 16872286 10.1111/j.1600-0684.2006.00171.x

[11] Dupinay T, Gheit T, Roques P, et al. Discovery of naturally occurring transmissible chronic hepatitis B virus infection among *Macaca fascicularis* from Mauritius Island. Hepatology. 2013;58(5):1610–1620. 23536484 10.1002/hep.26428

[12] Sa-Nguanmoo P, Rianthavorn P, Amornsawadwattana S, et al. Hepatitis B virus infection in non-human primates. Acta Virol. 2009;53(2):73–82. 19537907 10.4149/av_2009_02_73

[13] Liaw Y-F, Chu C-M. Hepatitis B virus infection. Lancet. 2009;373(9663):582–592. 19217993 10.1016/S0140-6736(09)60207-5

[14] Baird JK. Malaria zoonoses. Travel Med Infect Dis. 2009;7(5):269–277. 19747661 10.1016/j.tmaid.2009.06.004

[15] Liew JWK, Bukhari FDM, Jeyaprakasam NK, et al. Natural *Plasmodium inui* infections in humans and *Anopheles cracens* Mosquito, Malaysia. Emerg Infect Dis. 2021;27(10):2700–2703. 34545786 10.3201/eid2710.210412 PMC8462313

[16] Coatney GR, Chin W, Contacos PG, et al. *Plasmodium inui*, a quartan-type malaria parasite of old world monkeys transmissible to man. J Parasitol. 1966;52(4):660–663. 5969104

[17] Parr JWK. A guide to the large mammals of Thailand. Bangkok: Sarakadee Press; 2003.

[18] Aggimarangsee N. Survey of semi-tame colonies of macaques in Thailand. Nat Hist Bull Siam Soc. 1992;40(2):103–166.

[19] Malaivijitnond S, Hamada Y. Current situation and status of long-tailed macaques (*Macaca fascicularis*) in Thailand. Nat Hist J Chulalongkorn Univ. 2008;8:185–204.

[20] Perelygina L, Patrusheva I, Manes N, et al. Quantitative real-time PCR for detection of monkey B virus (Cercopithecine herpesvirus 1) in clinical samples. J Virol Methods. 2003;109(2):245–251. 12711069 10.1016/s0166-0934(03)00078-8

[21] Del Portillo P, Thomas MC, Martínez E, et al. Multiprimer PCR system for differential identification of mycobacteria in clinical samples. J Clin Microbiol. 1996;34(2):324. 8789008 10.1128/jcm.34.2.324-328.1996 PMC228790

[22] Heneine W, Switzer WM, Sandstrom P, et al. Identification of a human population infected with simian foamy viruses. Nat Med. 1998;4(4):403–407. 9546784 10.1038/nm0498-403

[23] Liu C, Chang L, Jia T, et al. Real-time PCR assays for hepatitis B virus DNA quantification may require two different targets. Virol J. 2017;14(1):94. 28494793 10.1186/s12985-017-0759-8 PMC5427580

[24] MacDonald DM, Holmes EC, Lewis JC, et al. Detection of hepatitis B virus infection in wild-born chimpanzees (*Pan troglodytes verus*): phylogenetic relationships with human and other primate genotypes. J Virol. 2000;74(9):4253–4257. 10756039 10.1128/jvi.74.9.4253-4257.2000 PMC111941

[25] Kamau E, Tolbert LS, Kortepeter L, et al. Development of a highly sensitive genus-specific quantitative reverse transcriptase real-time PCR assay for detection and quantitation of *Plasmodium* by amplifying RNA and DNA of the 18S rRNA genes. J Clin Microbiol. 2011;49(8):2946. 21653767 10.1128/JCM.00276-11 PMC3147742

[26] Snounou G, Viriyakosol S, Zhu XP, et al. High sensitivity of detection of human malaria parasites by the use of nested polymerase chain reaction. Mol Biochem Parasitol. 1993;61(2):315–320. 8264734 10.1016/0166-6851(93)90077-b

[27] Akter R, Vythilingam I, Khaw LT, et al. Simian malaria in wild macaques: first report from Hulu Selangor district, Selangor, Malaysia. Malar J. 2015;14(1):386. 26437652 10.1186/s12936-015-0856-3 PMC4595055

[28] Kumar S, Stecher G, Tamura K. MEGA7: molecular evolutionary genetics analysis version 7.0 for bigger datasets. Mol Biol Evol. 2016;33(7):1870–1874. 27004904 10.1093/molbev/msw054 PMC8210823

[29] Lee M-H, Rostal M, Hughes T, et al. Macacine herpesvirus 1 in long-tailed macaques, Malaysia, 2009–2011. Emerg Infect Dis. 2015;21(7):1107. 26080081 10.3201/eid2107.140162 PMC4480374

[30] Ohsawa K, Black DH, Torii R, et al. Detection of a unique genotype of monkey B virus (Cercopithecine herpesvirus 1) indigenous to native Japanese macaques (*Macaca fuscata*). Comp Med. 2002;52(6):555–559. 12540170

[31] Wisely SM, Sayler KA, Anderson CJ, et al. Macacine herpesvirus 1 antibody prevalence and DNA shedding among invasive rhesus macaques, Silver Springs State Park, Florida, USA. Emerg Infect Dis. 2018;24(2):345–351. 29350146 10.3201/eid2402.171439 PMC5782895

[32] Wilbur AK, Engel GA, Rompis A, et al. From the mouths of monkeys: detection of *Mycobacterium tuberculosis* complex DNA from buccal swabs of synanthropic macaques. Am J Primatol. 2012;74(7):676–686. 22644580 10.1002/ajp.22022 PMC3368330

[33] Rosenbaum M, Mendoza P, Ghersi BM, et al. Detection of *Mycobacterium tuberculosis* complex in new world monkeys in Peru. Ecohealth. 2015;12(2):288–297. 25515075 10.1007/s10393-014-0996-x PMC4470872

[34] Montali RJ, Mikota SK, Cheng LI. Mycobacterium tuberculosis in zoo and wildlife species. Rev Sci Tech. 2001;20(1):291–303. 11288517 10.20506/rst.20.1.1268

[35] Stenbak CR, Pinto-Santini DM, Murray SM, et al. Simian foamy viruses: infections in human and nonhuman primate hosts. In: Knauf S, Jones-Engel L, editors. Neglected diseases in monkeys: from the monkey-human interface to one health. Cham: Springer International Publishing; 2020.

[36] Ayouba A, Duval L, Liégeois F, et al. Nonhuman primate retroviruses from Cambodia: high simian foamy virus prevalence, identification of divergent STLV-1 strains and no evidence of SIV infection. Infect Genet Evol. 2013;18:325–334. 23612320 10.1016/j.meegid.2013.04.015

[37] Huang F, Wang H, Jing S, et al. Simian foamy virus prevalence in *Macaca mulatta* and zookeepers. AIDS Res Hum Retroviruses. 2012;28(6):591–593. 22236106 10.1089/AID.2011.0305

[38] Engel GA, Pizarro M, Shaw E, et al. Unique pattern of enzootic primate viruses in macaques. Emerg Infect Dis. 2008;14(7):1112–1115. 18598634 10.3201/eid1407.071643 PMC2600335

[39] Noppornpanth S, Haagmans BL, Bhattarakosol P, et al. Molecular epidemiology of gibbon hepatitis B virus transmission. J Gen Virol. 2003;84(Pt 1):147–155. 12533711 10.1099/vir.0.18531-0

[40] Seeger C, Mason WS. Hepatitis B virus biology. Microbiol Mol Biol Rev. 2000;64(1):51–68. 10704474 10.1128/mmbr.64.1.51-68.2000 PMC98986

[41] Fooden J. Malaria in macaques. Int J Primatol. 1994;15(4):573–596.

[42] Putaporntip C, Jongwutiwes S, Thongaree S, et al. Ecology of malaria parasites infecting Southeast Asian macaques: evidence from cytochrome b sequences. Mol Ecol. 2010;19(16):3466–3476. 20646216 10.1111/j.1365-294X.2010.04756.x PMC2921002

[43] Seethamchai S, Putaporntip C, Malaivijitnond S, et al. Malaria and Hepatocystis species in wild macaques, southern Thailand. Am J Trop Med Hyg. 2008;78(4):646–653. 18385364

[44] Amir A, Shahari S, Liew JWK, et al. Natural *Plasmodium* infection in wild macaques of three states in Peninsular Malaysia. Acta Trop. 2020;211:105596. 32589995 10.1016/j.actatropica.2020.105596

[45] Fungfuang W, Udom C, Tongthainan D, et al. Malaria parasites in macaques in Thailand: stump-tailed macaques (*Macaca arctoides*) are new natural hosts for *Plasmodium knowlesi, Plasmodium inui, Plasmodium coatneyi* and *Plasmodium fieldi*. Malar J. 2020;19(1):350. 33004070 10.1186/s12936-020-03424-0 PMC7528273

